# Co-operative Housekeeping

**Published:** 1890-09

**Authors:** 


### CO-OPERATIVE HOUSEKEEPING.

Theories are good, but practical demonstrations so much better, that we are glad to give our readers the benefit of the experience of some

Decatur (Ill.) householders, in their experiment with the co-operative housekeeping system. Fifty-two families of comfortable income banded together, a kitchen and necessary help was secured, with a paid housekeeper to oversee the cooking and serving. Then each housewife (of which there is one for each week of the year) takes her turn for a week in superintending the housekeeping—buying the supplies, arranging the *menu* for the week, keeping the accounts, etc. So far, all has worked like a charm : the husbands are satisfied, as the total cost for service and supplies, including all the luxuries of the season, is but $2.50 per week for each person ; the wives are equally well pleased, since it relieves them for fifty-one weeks out of the year, from all the housekeeper's cares and responsibilities, leaving them ample leisure for self-culture and the gratification of their individual tastes and ambitions, besides what is still more important, time to devote to the training and educating of their children. Of her newly acquired liberty and opportunities, one woman wittily says : In this way a woman can serve her week as head of the house, and take a trip around the world, if she wishes, before her turn to housekeep comes again." Verily, a new era has dawned for housekeepers and housekeeping.—S. I. M., in July *Good Health.*

